# Object-directed behaviors and human-directed sociability are linked in free-ranging dog puppies

**DOI:** 10.1016/j.isci.2025.113231

**Published:** 2025-07-29

**Authors:** Martina Lazzaroni, Giulia Cimarelli, Manon Delaunay, Friederike Range, Sarah Marshall-Pescini

**Affiliations:** 1 Domestication Lab, Konrad Lorenz Institute of Ethology, University of Veterinary Medicine Vienna, Savoyenstrasse 1a, 1160 Vienna, Austria; 2 Department of Chemistry, Life Science and Environmental Sustainability, University of Parma, Viale delle Scienze 17/A, 43124 Parma, Italy; 3 Behavioural Ecology Group, Wageningen University and Research, De Elst 1, 6708 WD Wageningen, the Netherlands; 4 Human Biology and Primate Cognition Group, Institute of Biology, Faculty of Life Sciences, Leipzig University, 04103 Leipzig, Germany

**Keywords:** Canine behavior, Biological sciences, Cognitive neuroscience

## Abstract

Most behavioral studies in dogs have investigated sociability and exploration of novelty as separate traits, often using different test settings and behavioral measures. However, this approach does not allow to assess whether these traits may instead reflect a shared underlying mechanism, such as a general tendency to approach novel stimuli, regardless of their social nature. In this study, we exposed pre-weaned free-ranging dog puppies to an unfamiliar human and a novel object. Free-ranging dog puppies were chosen to minimize the effect of experience and breed-selection biases. At 3 weeks of age, behaviors such as interaction, activity, and tail-wagging toward a novel object predicted similar behaviors toward a human, suggesting a unified trait rather than separate traits for sociability and object exploration. This challenges the traditional dichotomy between human-directed sociability and object exploration, proposing a common underlying mechanism. Nevertheless, puppies also showed more frequent behaviors with the social stimulus, likely due to greater sensitivity to animated stimuli.

## Highlights

• Early exploratory and social traits are consistent in free-ranging dog puppies
• At 3 weeks, sociability and exploration reflect a shared response to novelty
• The link between social and non-social traits weakens with age in dog pups

## Graphical abstract

**Figure:**
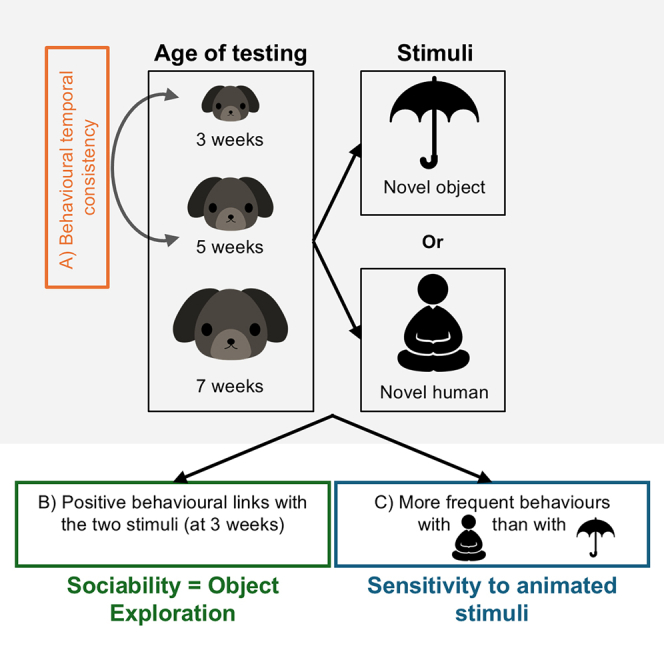

## Introduction

Several hypotheses emphasize the importance of changes in human-directed sociability allowing dogs to adjust to a human-dominated environment during dog domestication. For instance, tameness,^1^^,^^2^ hyper-sociability,^3^ and social competence,^4^ mostly toward humans, have all been suggested as possible candidates under selection during domestication. However, most studies aiming at testing such socio-specific domestication hypotheses have been conducted by comparing wolves’ and dogs’ behaviors toward unfamiliar humans, but rarely assessing subjects’ behavior toward non-social stimuli and thus controlling for their responses to novelty in general (exploration and neophobia),^5^ despite this being necessary to shed light onto the mechanisms that might have taken place during the process of domestication.

The link between non-social behaviors, such as exploration and neophobia, and sociability has already been suggested as an important aspect of domestication (see Behavioral Syndrome^6^^,^^7^ and Domestication Syndrome Hypothesis^1^^,^^2^). In support of this, studies conducted in large cohorts of pet dogs of different breeds found positive correlations between exploration in a non-social setting and sociability toward human strangers^8^ and conversely, a negative correlation between human-directed sociability and fearfulness toward novelty.^9^ Importantly, these associations were observed across different test contexts and behavioral measures, with sociability and exploration assessed separately in the same individuals. Such a link between social and non-social traits might not be restricted to domestic dogs, as also studies assessing personality traits in other wild species found positive correlations between exploration, activity, and conspecific sociability.^10^^,^^11^^,^^12^^,^^13^^,^^14^

However, the studies conducted on pet dogs have investigated sociability toward humans or conspecifics and exploration toward novelty separately, using different test settings and behavioral metrics.^8^^,^^9^ This leaves open the question of whether sociability and exploration are truly distinct traits or rather expressions of a shared underlying mechanism, such as a general tendency to approach novel stimuli, whether social or non-social.

Furthermore, to date, the few studies that have investigated the link between these behavior have mostly been conducted in adult pet dogs, where the effect of experience and breed may play a large role in shaping responses.^9^^,^^15^^,^^16^^,^^17^^,^^18^^,^^19^^,^^20^^,^^21^^,^^22^^,^^23^ To obtain clearer insights into the relationship between these behaviors and their relevance to domestication, it might be more informative to study a population that has not been subjected to intensive artificial selection for specific behaviors and to focus on developmental stages less influenced by learning (i.e., by testing puppies).

Free-ranging dogs are free to move, reproduce, and socially interact with each other and with other species, and thus still subject to the selective forces of the ongoing domestication process.^24^ In contrast to pet dogs, their behavior is neither affected by particular life experiences through contact with humans especially as puppies (i.e., no specific socialization, training, or living and testing conditions^25^) nor by artificial selection for specific phenotypic traits (as for breed dogs). Yet, humans might have a central role in the social interaction networks of free-ranging dogs, with interactions between the two species occurring frequently and taking on diverse forms over time.^26^ The degree and quality of the interactions can vary, dependent on the area in which dogs live (more or less urbanized or frequented by humans) as well as on the attitude that humans have toward free-ranging dogs in a given area.^27^^,^^28^

In the current study, we investigated the potential link between social and non-social traits in a population of free-ranging dog pups. We chose to test free-ranging dog puppies in the pre-weaning phase (at 3, 5, and 7 weeks)^29^^,^^30^ to keep the effect of experience on their behavior to a minimum, allowing us to test consistency in their behavioral responses across ages (important to be able to define these behaviors as part of temperament traits,^31^). In particular, we analyzed the link between the “exploration-avoidance” trait (sensu^31^), defined as an individual’s reaction to novelty or exploration of a new environment (measured by time spent interacting with a novel object), and the “human-directed sociability” trait, measured by time spent interacting with a human. Due to the constraints in the field, and the choice to test pups in a naturalistic setting (see in the following text), we could not include the classic measure of neophobia (time to approach the stimuli), since this was deemed unreliable in our context (i.e., group setting where the starting time was not the same for all subjects, see in the following text). We additionally investigated subjects’ activity levels (time spent moving around), arousal (in the form of tail-wagging duration), as well as fear and stress behaviors (e.g.^32^ and ^33^), which might provide a more comprehensive understanding of dogs’ reactions to novel objects and the humans.

We carried out a number of analyses to address our questions. First, we investigated the temporal consistency of the analyzed behaviors across ages, predicting that if they indeed capture an underlying temperament trait,^31^ a positive relationship between them should emerge. Second, in line with studies on wild species highlighting a correlation between conspecific sociability and exploration of novel stimuli^34^^,^^35^ and with studies on adult pet dogs showing a positive correlation between human-directed sociability and exploration of non-social stimuli (15–16), we predicted that behaviors elicited by a novel object would predict the occurrence and frequency of the same behaviors when puppies were exposed to an unfamiliar human, especially at a younger age, before differential experiences may affect the link.^36^ Finally, given that a difference in behavioral responses between the two contexts might not be of a kind, but of degree, we investigated whether subjects showed differences in the intensity of the expression of behavioral responses (e.g., tail-wagging and stress-related behaviors) between the two test conditions across ages.

## Results

### Behavioral consistency across ages

**Figure 1:**
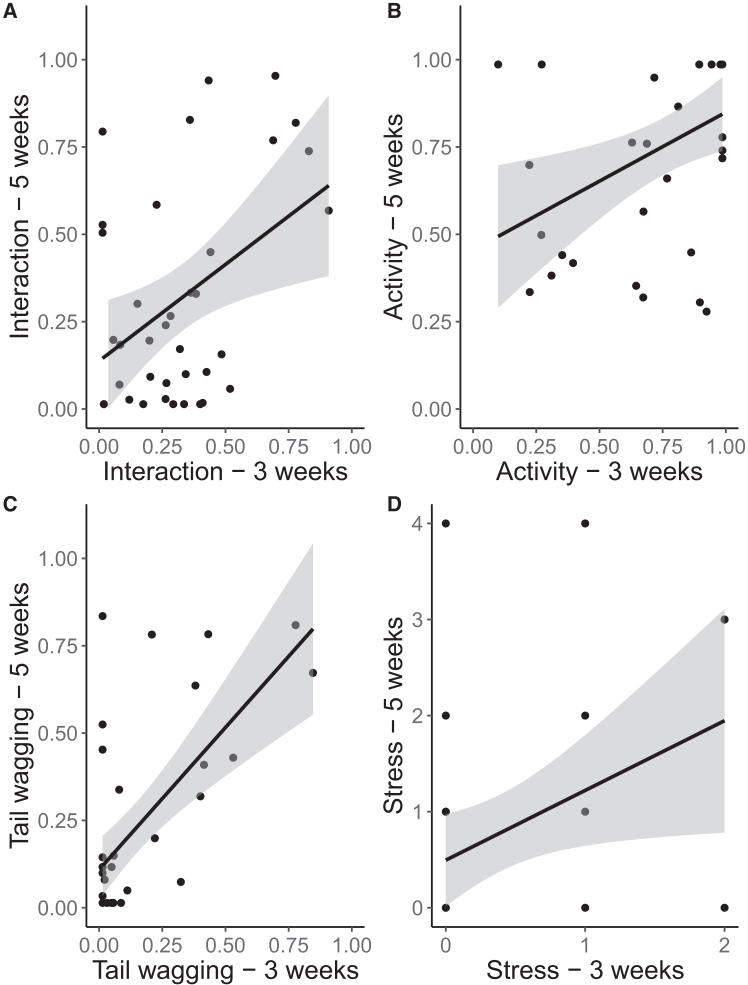
Behavioral consistency across ages (A–D) Proportion (A, B, and C) and frequency (D) of behaviors expressed toward both the unfamiliar human and the novel object, at 3 and 5 weeks (*x* and *y* axis, respectively): (A) interaction, (B) activity, (C) tail wagging, and (D) stress-related behaviors (count data, hence more than one individual is represented by a dot, if they showed the same number of stress-related behaviors).

For all behavioral variables tested, all null-full model comparisons resulted in significance (interaction: χ^2^ = 15.82, df = 3, *p* = 0.001; activity: χ^2^ = 13.86, df = 3, *p* = 0.003; tail wagging: χ^2^ = 20.41, df = 3, *p* < 0.001; stress-related behaviors: χ^2^ = 14.25, df = 3, *p* = 0.003). Independently from the type of stimulus (interaction at 3 weeks∗condition interaction: *p* > 0.05), puppies at 3 and 5 weeks interacted for a consistent proportion of time with the two stimuli (estimate ±SE = 1.71 ± 0.81, z-value = 2.12, *p* = 0.03, R-sq = 0.56). The same was true for activity (activity at 3 weeks∗condition interaction: *p* > 0.05; estimate ±SE = 1.36 ± 0.56, z-value = 2.42, *p* = 0.02, R-sq = 0.85), tail-wagging (tail-wagging at 3 weeks∗condition interaction: *p* > 0.05; estimate ±SE = 2.89 ± 0.86, z-value = 3.36, *p* = 0.001, R-sq = 0.61), and stress-related behaviors (Stress-related behaviors at 3 weeks∗condition interaction: *p* > 0.05; estimate ±SE = 0.94 ± 0.38, z-value = 2.45, *p* = 0.01, R-sq = 0.63) (Figure 1). No behavioral consistency emerged between 5 and 7 weeks (all null-full model comparisons: *p* > 0.05), see Data S1: complete statistical model outputs. Consistency of behaviors between 3 and 5 weeks was confirmed by intraclass correlation coefficients (ICC) measures (interaction: ICC = 0.38, *p* = 0.04; activity: ICC = 0.40, *p* = 0.02; tail-wagging: ICC = 0.41, *p* = 0.02; stress: ICC = 0.71, *p* < 0.001). ICC measures also revealed temporal consistency between 5 and 7 weeks for activity (ICC = 0.51, *p* = 0.005), tended to show some consistency for tail-wagging (ICC = 0.30, *p* = 0.08) but not for interaction (ICC = 0.26, *p* = 0.12) and stress (ICC = - 0.20, *p* = 0.81).

Significant results are summarized in Table 1 and full results are reported in Data S1: complete statistical model outputs.

**Table 1:** Summary of results

Research question	Response variable	Predictor (significant effect)
Behavioral consistency across ages	Interaction 5 weeks	- Interaction 3 weeks (+)
	Interaction 7 weeks	NS
	Activity 5 weeks	- Activity 3 weeks (+)
	Activity 7 weeks	NS
	Tail wagging 5 weeks	Tail wagging 3 weeks (+)
	Tail wagging 7 weeks	NS
	Stress-related behaviors 5 weeks	- Stress 3 weeks (+)
	Stress-related behaviors 7 weeks	NS
Links between object-directed and human-directed behaviors at different ages	Interaction social	3 weeks: Interaction object (+) 5 weeks: NS
	Activity social	3 weeks: Activity object (+) 5 weeks: NS
	Tail wagging social	3 weeks: Tail wagging object (+) 5 weeks: NS
	Stress-related behaviors social	3 weeks: NS 5 weeks: NS
Condition and age effects on puppies’ behaviors towards the stimuli	Interaction	Condition∗Age: 3 weeks: social > object 5 weeks: social > object 7 weeks: NS
	Activity	Condition∗Age: 3 weeks: social > object 5 weeks: social > object 7 weeks: NS
	Tail wagging	Condition: social > object Age: NS
	Stress-related behaviors	Age: 3w < 5w 5w > 7w Condition: NS

Relative to each research question, are reported for each response variable the significant predictors and their effect (+; >, <). NS indicates non-significant effect.

### Links between object-directed and human-directed behaviors at different ages

**Figure 2:**
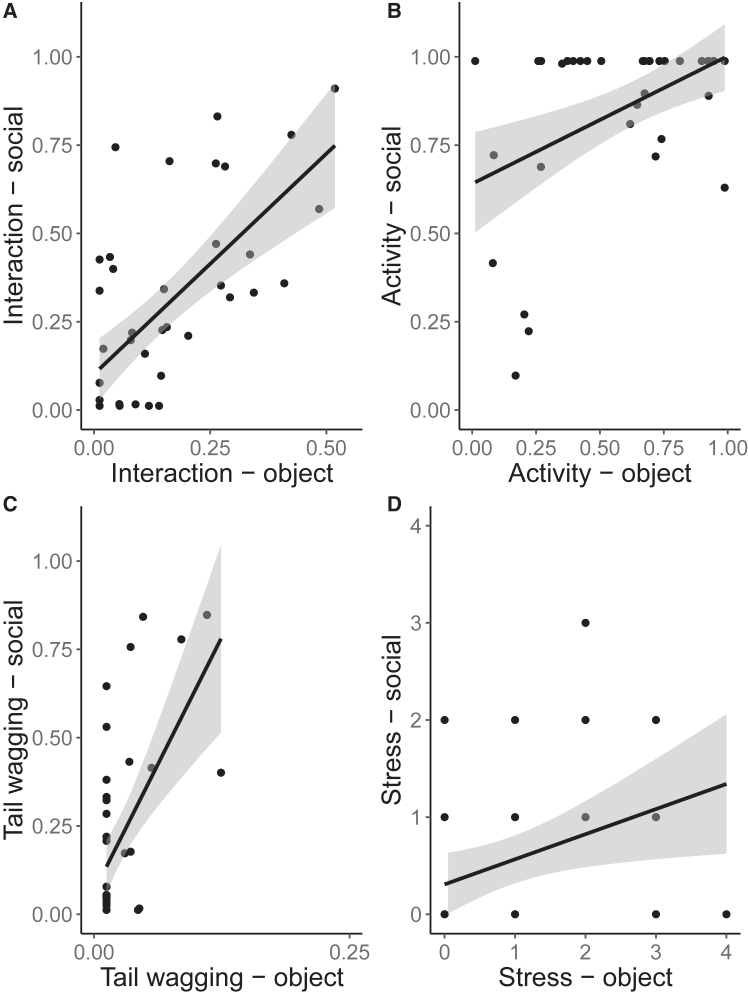
Link between object-directed and human-directed behaviors at three weeks of age (A–D) Proportion (A, B, and C) and frequency (D) of behaviors expressed toward the unfamiliar human and the novel object (*y* and *x* axis, respectively): (A) interaction, (B) activity, (C) tail wagging, and (D) stress-related behaviors (count data, hence more than one individual is represented by a dot, if they showed the same number of stress-related behaviors).

At 3 weeks, most behaviors shown during the novel object condition were significantly associated with the same behaviors shown in the unfamiliar human condition: the more the puppies interacted with the novel object, the more they interacted with the human (interaction, null-full model comparison: χ^2^ = 13.98, df = 1, *p* < 0.001; full model estimate: ±SE = 5.23 ± 1.34, z-value = 3.90, *p* < 0.001, R-sq = 0.73); the more they were active in the presence of the object, the more they were active in the presence of the human (activity, null-full model comparison: χ^2^ = 4.30, df = 1, *p* = 0.04; full model estimate ±SE = 1.31 ± 0.62, z-value = 2.13, *p* = 0.04, R-sq = 0.93); the more they wagged their tail when facing the object, the more they did so with the human present (tail-wagging, null-full model comparison: χ^2^ = 11.43, df = 1, *p* < 0.001; full model estimate ±SE = 22.71 ± 6.38, z-value = 3.56, *p* < 0.001, R-sq = 0.51). Only the model for stress-related behaviors was non-significant (null-full model comparison: χ^2^ = 1.23, df = 1, *p* = 0.27) (Figure 2). None of the behaviors shown in the novel object test had a predictive value for the behaviors exhibited toward the unfamiliar human at 5 weeks (all null-full model comparisons: *p* > 0.05), see Data S1: complete statistical model outputs.

### Condition and age effects on puppies’ behaviors toward the stimuli

**Figure 3:**
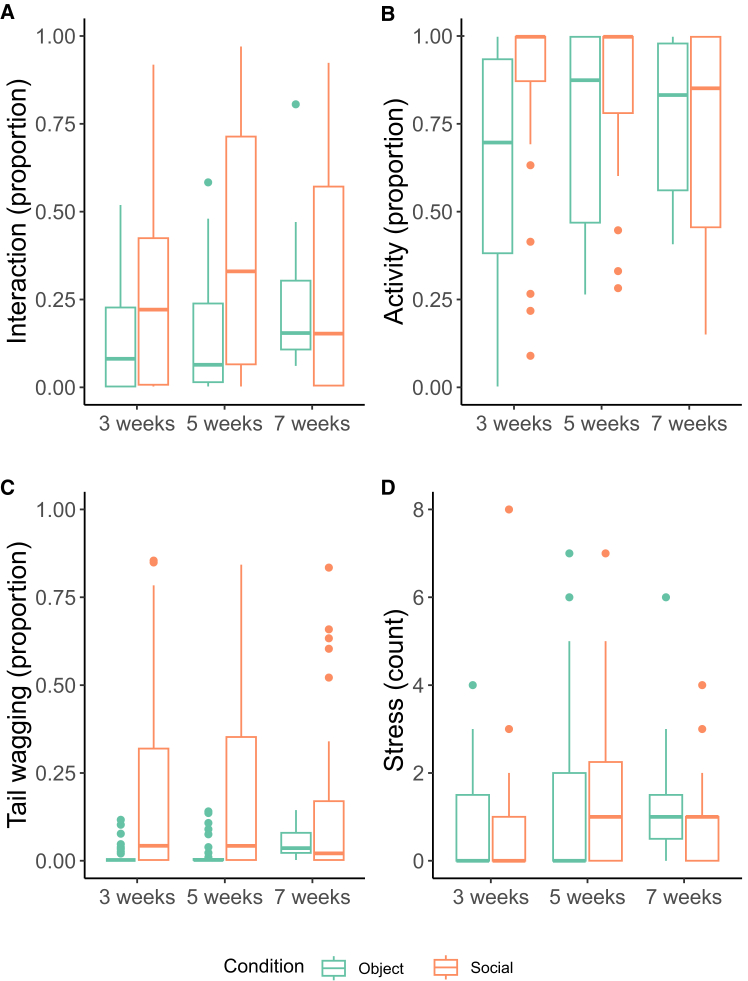
Expression of behaviours across ages in both test conditions Proportion (A, B, and C) and count (D) of behaviors expressed by puppies at 3, 5, and 7 weeks of age in the object and in the human condition. (A) Interaction, (B) activity, (C) tail wagging, and (D) stress-related behaviors.

The null-full model comparison for the variable interaction was significant (χ^2^ = 25.84, df = 5, *p* < 0.001), showing a significant effect of the interaction between age and condition (χ^2^ = 10.25, df = 2, *p* = 0.006). In particular, we found that puppies at both 3 and 5 weeks, but not at 7 weeks, interacted more with the unfamiliar human than with the object (3 weeks: estimate ±SE = - 0.50 ± 0.22, z-value = - 2.31, *p* = 0.02; 5 weeks: estimate ±SE = - 1.24 ± 0.27, z-value = - 4.54, *p* < 0.001; 7 weeks: estimate ±SE = 0.31 ± 0.41, z-value = 0.75, *p* = 0.45; full model R-sq = 0.47). A similar pattern emerged for the variable activity (null-full model comparison: χ^2^ = 31.29, df = 5, *p* < 0.001; condition∗age interaction: χ^2^ = 6.98, df = 2, *p* = 0.03): puppies at 3 and 5 weeks were more active in the presence of the unfamiliar human than when tested with the novel object (3 weeks: estimate ±SE = −1.14 ± 0.23, z-value = - 4.88, *p* < 0.001; 5 weeks: estimate ±SE = - 0.59 ± 0.26, z-value = - 2.27, *p* = 0.02; 7 weeks: estimate ±SE = 0.05 ± 0.41, z-value = 0.12, *p* = 0.90; full model R-sq = 0.85). Puppies also wagged their tail for longer in the human than in the novel object condition (null-full model comparison: χ^2^ = 28.28, df = 5, *p* < 0.001; reduced model, social vs. object: estimate ±SE = 0.84 ± 0.16, z-value = 5.13, *p* < 0.001, R-sq = 0.29), independent of age (condition∗age interaction and age, both *p* > 0.05). Stress-related behaviors (null-full model comparison: χ^2^ = 22.43, df = 5, *p* < 0.001), were shown by puppies more at 5 weeks than both at 3 and at 7 weeks (reduced model, 5 weeks: estimate ±SE = 0.50 ± 0.21, z-value = 2.36, *p* = 0.048; 7 weeks: estimate ±SE = 0.88 ± 0.26, z-value = 3.36, *p* = 0.002; R-sq = 0.60), independently from condition (condition∗age and condition, both *p* > 0.05) (Figure 3), see Data S1: complete statistical model outputs.

## Discussion

With the current study, we investigated the consistency across ages of the behavioral responses toward novel objects and unknown people in free-ranging dog pups, tested the potential link between these, and evaluated whether qualitative and quantitative differences would emerge in the puppies’ behavioral responses in the two contexts.

At the individual level, regardless of the test condition, subjects consistently spent similar amounts of time interacting, being active, wagging their tails and performing stress-related behaviors both in the presence of the human and the object between three and five weeks of age but not between five and seven weeks of age. The evidence for consistency of these behavioral responses observed during the early stages of life (at three and five weeks) suggests they may indeed reflect temperament-related traits.^7^^,^^31^^,^^37^ The lack of consistency between five and seven weeks of age could be due to the reduced number of tested subjects at seven weeks, or, as observed in various species and for different behavioral traits,^38^^,^^39^ it may be due to the fact that as individuals mature and/or gain more life experiences, their behavior changes. Indeed, in addition to genetic factors, environmental factors during early development significantly impact personality formation,^39^^,^^40^ allowing individuals to adjust to their specific environment. In fact, dogs do demonstrate an exceptional ability to adapt to human-dominated environments, with examples of them transitioning from free-ranging dogs with varying degrees of human association (from village dogs to feral dogs), to becoming human companions, adjusting to diverse living conditions, even within a single lifetime.^27^

Regarding our second research question, clear links between the pups’ behavioral responses toward the object and the human emerged at an early age. At 3 weeks of age, most behavioral responses expressed toward a novel object (i.e., interaction time, activity, and tail wagging but not stress-related behaviors) predicted similar responses toward the unknown human. In most previous studies, these behaviors are considered either as a measure for the subjects’ exploration/interest toward an object^41^^,^^42^ or as a measure of human-directed sociability^43^ depending on the test set-up. However, our results suggest that rather than being separate components, responses to the novel object and the unfamiliar human may in fact be viewed (at least at 3 weeks) as the expression of a single trait i.e., the subject’s overall exploratory tendency and general responsiveness to novel stimuli. These results are in line with those from a number of other species where a positive correlation between exploration, activity and conspecific sociability have been found,^10^^,^^11^^,^^12^^,^^13^ as well as studies on adult pet dogs of different breeds where correlations emerged between exploration of non-social stimuli and sociability toward unknown humans.^8^^,^^9^ These results support the hypotheses according to which evolutionary processes such as domestication might have an effect on a set of behavioral traits, potentially genetically linked to one another (i.e., behavioral syndrome^7^), or that a common temperament trait might explain behavioral reactions to both inanimate and animate stimuli.

Such link was not present at five weeks though (when sample size was still adequate). A reason for this might be due to the effect of experience, potentially differentiating between the two stimuli in a short period of time (i.e., the two weeks between the 3- and 5-week-old tests). In fact, in our study, we observed exploration already at three weeks of age. Dogs’ motivation to explore novel stimuli has been reported to start very early in dogs’ ontogeny, starting at birth with a primary period when sensory acuity is developing^44^^,^^45^ and with a significant transition from three/four weeks when their vision improves,^29^^,^^30^^,^^45^ and lasting up to 16 weeks of age.^29^^,^^46^ Such an early exposure from birth with a complex, anthropic environment might have allowed dogs to experience and adapt to their surroundings, shaping their behavioral responses. In particular, as these village dogs den in humans’ vicinity,^47^ puppies might have extensive possibilities of interacting with humans and objects, already starting at 3 weeks, when they emerge from their dens. Although puppies of the studied population are also exposed to both types of stimuli, since the surroundings of their dens are usually littered with rubbish, they might have learned through direct (e.g., people providing food) or indirect (i.e., through the observation of the mother interacting with people) that humans might be more relevant than inanimate objects. Consequently, the perceptual and motor development, as well as life experiences of the subjects, likely play a crucial role in shaping their behavioral responses during their early ontogeny,^48^ even between three and five weeks of age, resulting in the absence of evidence for a link between the behaviors shown toward the object and the human. We could not investigate whether such link could present again at a later age, as at 7 weeks, a limited sample size did not allow a similar analysis.

An alternative explanation for a link at 3 weeks, but not at 5 weeks, is that at 3 weeks, puppies did not differentiate between the human and the object. However, dog puppies at 3 weeks of age already have a fully developed olfaction and they can already see and hear (although not perfectly^30^), which was confirmed by our results showing that puppies could distinguish between the two test conditions both at 3 and 5 weeks, with subjects exhibiting behavioral responses (i.e., interaction, activity, and tail wagging) for longer toward the human than the object. These findings might be due to an interplay between different factors. Firstly, as suggested by the hyper-sociability hypothesis,^3^ these results may support the view that domestication has heightened dogs’ need for social contact, including with heterospecifics. Another possible simpler explanation is that the human may have represented a more salient stimulus compared to the object because she was moving and talking,^49^ as already observed in adult dogs,^50^ since animate stimuli could be more attention-grabbing than inanimate ones due to domain-specific mechanisms (animate monitoring hypothesis,^51^). For example, a recent neuroimaging study comparing dogs and humans found functionally analogous body- and animacy-responsive areas in the occipito-temporal lobe of both humans and dogs.^52^ Furthermore, preference for face-like stimuli have been found in newly hatched chicks,^53^ new-born primate^54^ and even in solitary-living tortoise hatchlings,^55^ suggesting another aspect potentially affecting our dog puppies’ differential reaction to the human stimuli. Future studies could investigate whether indeed social stimuli attract more attention and are preferred by dog puppies by presenting two or more stimuli at the same time, rather than on different days (similarly to Gácsi et al.^56^).

Interestingly, differences between conditions did not hold at 7 weeks. The reduced number of individuals at 7 weeks might have strongly influenced these results. Nevertheless, it is noteworthy that the difference in tail wagging was not modulated by age. The complexity of dogs’ tail wagging, with its multifaceted functions such as social signaling, expressing positive emotions, requesting signals, or indicating arousal (for a review refer the study by Leonetti et al.^57^), makes it challenging to pinpoint its exact functions. In our case, the occurrence of tail wagging in both social and asocial conditions suggests that, in this test, it may have been driven, at least partially, by a general increase in subjects’ arousal, particularly in response to the presence of the human. Additionally, its potential communicative function could explain why tail wagging remained consistently higher in the social condition compared to the object condition across all test ages.

Taken together, results suggest that both the exploration of non-social stimuli and human-directed sociability are stable traits in free-ranging dog pups, but with increasing age, the positive correlation link between them decreases, likely due to the modulating effect of experience. This behavioral plasticity coupled with the variation/extension of dogs’ critical period of socialization^29^^,^^30^ enables dogs to adjust their responses to varying human environments based on their different life experiences, further enhancing their ability to succeed in such contexts.^48^

The complexity of sociability likely requires multifaceted measures^43^ to capture its various components, thus, future studies should investigate the link between exploration in the non-social setting and different aspects of sociability (e.g., time spent in proximity, latency to approach, frequency of social behaviors, attractiveness toward others, tolerance, etc.). This might be particularly relevant when considering that in non-domestic species, humans are generally used as stimuli to measure an animals’ boldness (e.g., flight initiation test^31^), rather than their “sociability”, since humans are assumed to be a potential threat (comparable to a predator) for most wild species.^31^^,^^58^ Here, we speculate that although the history of domestication may have changed how dogs consider humans (i.e., as potential social partners), human-directed interactions might still be linked to an overall low level of fearfulness, at least as pups, before extensive direct experience with humans modulates their response. This would result in an increased likelihood of approaching and ultimately exploring both new objects/situations and unfamiliar humans, and it might have been essential for dogs to thrive in a human-dominated environment,^59^ mirroring recent findings of wild animal populations adapting to urbanized areas.^60^^,^^61^ Analyses relating to all three aspects i.e., conspecific sociability, human-directed sociability, and exploration in a non-social context are still needed, to fully understand the interplay between these traits.

### Limitations of the study

A key limitation of this study is the reduced sample size at 7 weeks, which may have impacted the reliability of findings for this age group. Additionally, the use of a static, inanimate object as a novelty stimulus may not fully capture the range of exploratory behaviors that might be elicited by a more dynamic, moving object, which could provide a better control for assessing novelty responses. Furthermore, the behavioral measures used, such as time spent interacting and tail-wagging, may not fully encompass the complexities of sociability and exploration, suggesting that a broader range of behaviors and more nuanced measures might yield a more comprehensive understanding. In line with this, having tested puppies in a group setting, despite providing the advantage of a naturalistic scenario, limited the possibility of measuring behaviors such as the latency to approach. Lastly, the absence of longitudinal data beyond early ontogeny restricts insight into how these early behaviors and traits may evolve or stabilize into adulthood, indicating a need for longer-term studies to better understand the persistence and stability of these traits over time.

## Resource availability

### Lead contact

Further information and requests for data and code should be directed to and will be fulfilled by the lead contact, Dr. Martina Lazzaroni (martina.lazzaroni@unipr.it).

### Materials availability

This study did not use newly generated materials or reagents.

### Data and code availability

• The raw data are provided in Data S2: raw data.
• Any additional information required to reanalyze the data reported in this paper is available from the lead contact upon request.

## Acknowledgments

We would like to thank the local authorities of the Sous-Massa region and Prof. Ikhlass El Berbri of the University Hassan II (Rabat, Morocco) for their support. Moreover, we would like to thank Larissa Darc (LD), Luca Secker (LS), Klaudia Tondos (KT), Rachel Dale (RD), Elizabeth Baxter (EB), Juliette Gratalon (JG), Kiah Buddington (KB), Adrienne Guignard (AG), and Andreas Berghänel (AB) for their help with data collection. We thank Ursa Blenkus for coding the videos for inter-rater reliability. This research was funded by the DOC fellowship of the Austrian Academy of Sciences and by the Austrian Science Fund (10.13039/501100002428FWF) “DK Cognition and Communication 2”: W1262-B29 [10.55776] to ML, the Austrian Science Fund (10.13039/501100002428FWF) projects P-34749 to G.C., P-34675 to F.R. and I-5052 to S.M.-P. and the VetMedUni Start-Up grant to G.C. For open access purposes, the authors have applied a CC-BY public copyright license to any author-accepted manuscript version arising from this submission. The photograph in Figure 1 was captured by Martina Lazzaroni.

## Author contributions

Conceptualization, M.L., S.M.-P, and F.R.; data curation, M.D., G.C., and M.L.; formal analysis, G.C.; funding acquisition, M.L., S.M.-P., F.R., and G.C.; investigation, M.L. and G.C.; methodology, M.L., S.M.-P., F.R., G.C., and M.D.; project administration, M.L., S.M.-P., and G.C.; visualization, G.C.; writing-original draft, M.L., G.C., and S.M.-P.; writing-review and editing, M.L., G.C., S.M.-P., F.R., and M.D.; all authors revised and approved the manuscript.

## Declaration of interests

The authors declare no competing interests.

## STAR★Methods

### Key resources table

**Table:** 

REAGENT or RESOURCE	SOURCE	IDENTIFIER
**Experimental model: Organisms/strains**		

Free-ranging dog puppies (*Canis lupus familiaris*)	Taghazout and Tamraght, Agadir, Morocco	Field study population; see STAR Methods

**Software and algorithms**		

BORIS (Behavioral Observation Research Interactive Software)	Friard & Gamba,^62^ 2016; https://www.boris.unito.it/	Version used not specified; open source
R (statistical computing environment)	R Core Team; https://www.r-project.org/	Version 4.3.1
glmmTMB R package	Brooks et al.,^63^ 2017; CRAN	https://cran.r-project.org/package=glmmTMB
ggplot2 R package	Wickham, 2016^64^; CRAN	https://ggplot2.tidyverse.org/
emmeans R package	CRAN	https://cran.r-project.org/package=emmeans
irrNA, Irr, Lpsolve, stats R packages	CRAN	Used for reliability/statistics

**Other**		

Raw data (Data S2)	This paper (Supplemental File Sets)	Data S2
Statistical model outputs (Data S1)	This paper (Supplemental File Sets)	Data S1
Novel objects (umbrella, suitcase, cardboard, bucket)	Commercially available	Used as novel stimuli; see STAR Methods
Human experimenter (social stimulus)	This paper (study team)	See STAR Methods for procedure

### Experimental model and study participant details

Free-ranging dog puppies were tested in their natural environment in the municipalities of Taghazout and Tamraght, Agadir, Morocco where a Domestication Lab field site was established by Priv. Doz. Dr. Marshall-Pescini, Prof. Dr. Range and Dr. Lazzaroni in 2016. The experiments were conducted for 3 years, between 2017 and 2020 by 10 experimenters (JG, LB, KB, GC, RD, ML, AG, LS, KT, CB, see Acknowledgments). The study area was monitored daily by team members as part of the general data collection procedure of the dog population. Whenever a pregnant or lactating female was observed, the team members would try to follow her to identify the den. Only puppies whose birth date was known were included in the study. As previously observed,^47^ free-ranging dogs’ dens were usually located in areas of high human activity, close to human-related food resources. Dens were settled inside cavities such as those found in piles of branches, rocks or construction material, but also in less protected spots such as among piles of beach loungers. The proximity of dens to human activities determines puppies’ early exposure to artificial objects and noises, as well as humans. This typically begins when the puppies start to walk (three weeks of age) moving outside and around the den.

Free-ranging dog puppies from 14 litters were involved in the study (*n* = 105 at 3 weeks, *n* = 73 at 5 weeks, and *n* = 48 at 7 weeks of age). The decreasing number of individuals across ages is due to the death or disappearance of subjects over time. The number of individuals tested per condition also differed since participation in the test was voluntary and not all individuals were awake or came out of the den during testing (see table below). Each subject tested was photographed and added to a database consisting of individual ID, litter, sex, age, and pictures allowing us to identify the same individual over time. See for information related to the distribution of sex and age of each participant in the file Data S2: raw data.

**Table:** Number of tested subjects at each age and in each condition

Age	Condition	Number of tested subjects (females, males)
3 weeks	Object Social	*n* = 47 (*n* = 23 females, *n* = 20 males, *n* = 2 sex unknown) *n* = 49 ( *n* = 26 females, *n* = 21 males, *n* = 2 sex unknown)
5 weeks	Object Social	*n* = 35 (*n* = 24 females, *n* = 10 males, *n* = 1 sex unknown) *n* = 44 (*n* = 27 females, *n* = 16 males, *n* = 1 sex unknown)
7 weeks	Object Social	*n* = 11 (*n* = 7 females, *n* = 4 males) *n* = 29 (*n* = 17 females, *n* = 12 males)

#### Ethical statement

The authorization to test the free-ranging dogs was provided by the municipality of Taghazout (Morocco). Ethical approval for this study was obtained from the Ethical Committee for Animal Veterinary Science and Public Health of the University Hassan II Rabat, Morocco (Protocol number: CESASPV_2023_04). All procedures applied in the present study were noninvasive and in accordance with the European Union Directive on the protection of animals used for scientific purposes (EU Directive 2010/63/EU).

### Method details

#### Experimental design

The stimuli were presented in front of the den, to the whole litter, thus allowing littermates to express their natural behaviors without prior potentially stressful experience of being picked up and separated from their littermates (see also Johnson-Ulrich et al.^65^ for group testing in the wild).

The mother was called away with a bit of food by one of the experimenters while the pups were being tested. Either a seated human (i.e., social condition) or an object (i.e., novel object condition) was presented 1–2 m in front of the den to the whole litter for 5 minutes (see below figure). In the social condition, the experimenter sat cross-legged quietly without initiating contact with the subject. For the novel object condition, a large object (i.e., umbrella, suitcase, cardboard measuring 1m–1.5m, a bucket) was placed in front of the den. The two conditions were presented on two consecutive days at three age periods: 3, 5 and, 7 weeks, for a total of 6 trials. Across ages, the experimenters and the novel object differed, so that the same litter never saw the same person/object more than once. The order of the conditions (social vs. novel object) was counterbalanced at each age and across litters. All tests were video recorded, with a recorder placed on a tripod within the testing area.

**Figure:**
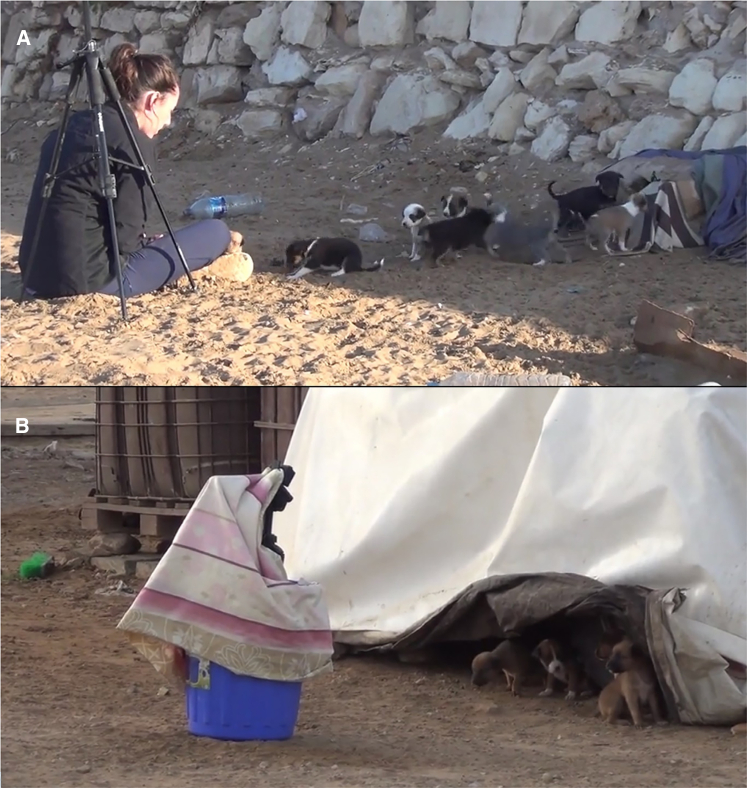
Test setting and procedure (A and B) The stimulus, either a human (A) or a novel object (B) is placed in front of the den for 5 min.

#### Behavioral coding

The video of each test was watched multiple times, so that every pup’s behavior was coded using continuous focal animal sampling. The test started when the stimulus was in position and lasted a maximum of 5 minutes. However, the total duration of the test could be different for each pup since the actual start of the test for each pup was defined as the pup looking at the stimulus for the first time. In 17 cases, the focal pup was already approaching or interacting with the object at the start of the video. Hence, in these cases, the start of the test coincided with the start of the video. Considering that the distance from the stimulus varied across subjects (i.e., it depended on the first glance at the stimulus, which could have happened from different locations), it was not possible to measure the latency to interact with the stimulus in a comparable way across subjects. We designed an ethogram and coded behaviors related to exploration and positive arousal (proportion of time interacting with the stimulus, proportion of time being active, proportion of time wagging the tail, number of play bows), the number of aggressive behaviors (bared teeth, growling, piloerection), behaviors related to fear (proportion of time with the tail tucked in, proportion of time with a hunched posture, number of startle responses), and behaviors previously related to stress/negative arousal (number of paw lifts, number of nose/lips licks, number of yawns, number of head/body shakes, proportion of time spent self-grooming, and number of scratching bouts). See table below for definitions. However, many of the behaviors initially outlined in our ethogram did not occur (e.g., aggressive, tucked tail, hunched posture) or occurred in too few instances (i.e., play bow, startle response, paw lift, nose/lips licking) to be included in the statistical analyses. The number of yawning, head/body-shaking, self-grooming, and scratching bouts were summed up in a variable called “Stress-related behaviors”. Videos were coded using the Software BORIS.^62^

**Table:** Ethogram of the coded behaviors including their detailed definition, the type of variable used in the analyses, and the number of subjects showing the behavior across all test trials

Behavior	Definition	Type of variable	No. of subjects showing the behavior across all test trials
Interaction with stimulus	Contact with the stimulus using nose, mouth, tongue (licking) or paws, including climbing on the stimulus.	Proportion of time	*N* = 170
Activity	Standing on four legs, walking, trotting, running	Proportion of time	*N* = 215 (all)
Tail wagging	Rhythmic repeated tails movement while approaching or interacting with the stimulus.	Proportion of time	*N* = 105
Play bow	The forelimbs are bent down while the hind legs are raised; the tail is held high.	Count	*N* = 8 (2 object, 6 social)
Aggressive	Sum of the following: bared teeth, growling, piloerection	Count	*N* = 0
Tucked tail	The tail is positioned below the body midline, between the hind legs.	Proportion of time	*N* = 0
Hunched posture	The back is curved upward, the head lowered toward the ground.	Proportion of time	*N* = 0
Startle	Quick, sharp movement, such as a little jump with the contraction of the whole body.	Count	*N* = 8 (6 object, 2 social)
Paw lift	One front paw is lifted off the ground and slightly bent	Count	*N* = 0
Nose/lips licking	Tongue runs over the nose/lips	Count	*N* = 1 (object)
Yawning	The mouth is wide open while the eyes are closed.	Count	*N* = 21 (11 object, 10 social)
Head/body shaking	Quick half-rotations of the head/body resulting in the shudder of the head/body.	Count	*N* = 54 (25 object, 29 social)
Self-grooming	The subject nibbles or licks themselves.	Count	*N* = 35 (12 object, 23 social)
Scratching	The subject moves one of the paws to touch a specific part of the body in a quick repetitive way	Count	*N* = 40 (15 object, 25 social)

#### Inter-rater reliability

To assess the inter-rater reliability of data coding, a second observer (UB) coded 15% of randomly selected videos (i.e., *N* = 90 replicates were coded). IntraClass Correlation coefficients (ICC) were calculated using the Irr and Lpsolve package and were acceptable for all behaviors (Interaction: ICC = 0.96, F(179, 179) = 47.5, *p* < 0.001; Activity: ICC = 0.89, F(89, 89.8) = 17.6, *p* < 0.001; Tail wagging: ICC = 0.67, F(89, 81.1) = 5.24, *p* < 0.001; Stress-related behavior: ICC = 0.86, F(359,336) = 13.1, *p* < 0.001).

### Quantification and statistical analysis

To test whether the behaviors shown during the test were consistent across ages, and hence could be the expression of an underlying temperament trait,^31^ we ran two sets of models, with the proportion of time spent interacting with the stimulus, the proportion of time spent active, and the proportion of time spent tail-wagging (GLMMs with beta distribution, function *glmmTMB* of the package glmmTMB), as well as with the number of stress-related behaviors (GLMMs with poisson distribution, function *glmmTMB* of the package glmmTMB) as response variables, either shown at 5 weeks (with the same variable shown at 3 weeks as predictor, *N* = 36), or at 7 weeks (with the same variable shown at 5 weeks as predictor, *N* = 29). In both cases, we included age in interaction with condition as the temporal consistency of the analyzed behaviors could have been dependent on the stimulus the subjects were exposed to. The random effect part contained the identity of the subject nested within litter. A null model, not containing the two-way interaction but otherwise identical to the full model, was also built and compared to the full model to reduce the likelihood of committing a Type 1 error. The null-full model comparison was conducted using the *anova* function (Chisquare test, package stats). When the null-full model comparison resulted as significant, we investigated the significance of the test predictors using the *drop1* function (Chisquare test, package stats). When the interaction was significant, we analyzed the differences between each level of the variables included in the interaction using the *emmeans* function (emmeans package), correcting for multiple testing using the Tukey method. When the interaction was non-significant, we ran a reduced model with age as main effects and analyzed its significance using the *drop1* function. For this set of models, we disregarded a potential main effect of condition as this was redundant with the second set of models (see above). Estimates were extracted using the *summary* function. Additionally, we measured ICC values for each behavioral variable to assess temporal consistency between 3 and 5 weeks, as well as between 5 and 7 weeks using the function iccNA from the package irrNA.

To analyze whether the behaviors shown toward the novel object were predictive of the behaviors shown toward the unfamiliar human, we ran two sets of models with the proportion of time spent interacting with the stimulus, the proportion of time spent active, and the proportion of time spent tail-wagging (GLMMs with beta distribution, function *glmmTMB* of the package glmmTMB,^63^ as well as with the number of stress-related behaviors (GLMMs with poisson distribution, function *glmmTMB* of the package glmmTMB^63^) shown during the human conditions as response variables, separately at 3 weeks (*N* = 42) and at 5 weeks (*N* = 29). The corresponding behavioral variable exhibited during the novel object condition was included as test predictor (thus for example, the time spent interacting with the *object* was entered as predictor variable where the time spent interacting with the *human* was the response variable). The number of puppies in the litter was included as a control variable and the identity of the litter as a random factor. Also in this case, a null model, not containing the test predictor but otherwise identical to the full model, was also built and compared to the full model to reduce the likelihood of committing a Type 1 error.^66^^,^^67^ The null-full model comparison was conducted using the *anova* function (Chisquare test, package stats). When the null-full model comparison was significant, we investigated the significance of the test predictor using the *drop1* function (Chisquare test, package stats.^68^ We analyzed the dataset separately for each week because not all subjects participated in both conditions at all ages, hence this approach allowed us to keep a larger sample size. As only *N* = 10 puppies participated in both conditions at 7 weeks of age, we only ran the analyses at 3 and at 5 weeks.

Finally, to test whether pups behaved differently toward the person and the object at the different ages we ran a set of models with the proportion of time spent interacting with the stimulus, the proportion of time spent active, and the proportion of time spent tail-wagging (GLMMs with beta distribution, function *glmmTMB* of the package glmmTMB), as well as with the number of stress-related behaviors (GLMMs with poisson distribution, function *glmmTMB* of the package glmmTMB) as response variables. In all models, we included the two-way interaction between condition and age as test predictors as well as the number of puppies in the litter as a control variable (to control for the fact that a subject could be distracted by the other puppies and that subjects in more numerous litters would have higher chances of being distracted). The identity of the subject nested within litter were included as random effects. A null model, not containing the two-way interaction nor the test predictors involved in it, but otherwise identical to the full model, was also built and compared to the full model to reduce the likelihood of committing a Type 1 error. The null-full model comparison was conducted using the *anova* function (Chisquare test, package stats). When the null-full model comparison was significant, we investigated the significance of the test predictors using the *drop1* function (Chisquare test, package stats). When the interaction was significant, we analyzed the differences between each level of the variables included in the interaction using the *emmeans* function (emmeans package), correcting for multiple testing using the Tukey method. When the interaction was non-significant, we ran a reduced model with condition and age as main effects and analyzed their significance using the *drop1* function. Estimates were extracted using the *summary* function. The effect of sex was not analyzed as not part of our main research question and to avoid to increase model complexity. Information related to the sex of the subject are reported in Data S2: raw data. The full statistical outputs can be found in Data S1: complete statistical model outputs.

All plots were made using the function *ggplot* from the package ggplot2. All analyses were conducted in R (R Core Team, version 4.3.1) and the significance threshold was set at α = 0.05.

## Supplemental information

Data S1. Complete statistical model outputs

Data S2. Raw data
